# Hemostatic and antibacterial PVA/Kaolin composite sponges loaded with penicillin–streptomycin for wound dressing applications

**DOI:** 10.1038/s41598-021-82963-1

**Published:** 2021-02-09

**Authors:** Tamer M. Tamer, Maysa M. Sabet, Ahmed M. Omer, Eman Abbas, Alaa I. Eid, Mohamed S. Mohy-Eldin, Mohamed A. Hassan

**Affiliations:** 1 Polymer Materials Research Department, Advanced Technology and New Materials Research Institute (ATNMRI), City of Scientific Research and Technological Applications (SRTA-City), New Borg El-Arab City, P.O. Box: 21934, Alexandria, Egypt; 2 Zoology Department, Faculty of Science, Alexandria University, Alexandria, Egypt; 3 Advanced Materials Division, Composites Department, Central Metallurgical Research Institute (CMRDI), Eltebbin, Helwan, 12422 Cairo Egypt; 4 Protein Research Department, Genetic Engineering and Biotechnology Research Institute (GEBRI), City of Scientific Research and Technological Applications (SRTA-City), New Borg El-Arab City, P.O. Box: 21934, Alexandria, Egypt

**Keywords:** Biomedical materials, Antimicrobials, Biopolymers

## Abstract

Hemorrhage is the major hindrance over the wound healing, which triggers microbial infections and might provoke traumatic death. Herein, new hemostatic and antibacterial PVA/Kaolin composite sponges were crosslinked using a freeze-thawing approach and boosted by penicillin–streptomycin (Pen-Strep). Physicochemical characteristics of developed membranes were analyzed adopting Fourier transformed infrared spectroscopy (FT-IR), scanning electron microscopy (SEM), a thermal gravimetric analyzer (TGA), and differential scanning calorimetry (DSC). Furthermore, the impacts of kaolin concentrations on porosity, swelling behavior, gel fraction, and degradation of the membranes were investigated. SEM analyses revealed a spongy-like structure of hydrogels associated with high dispersion of kaolin inside PVA matrix. The thermal characteristics of PVA/Kaolin were significantly ameliorated compared to the prime PVA. Moreover, the results exhibited significant variations of swelling performance, surface roughness and pore capacity due to the alterations of kaolin contents. Besides, the adhesive strength ability was manifestly enhanced for PVA-K0.1 sponge. Biomedical evaluations including antibacterial activity, blood clotting index and thrombogenicity of the membranes were studied. The contact of PVA/Kaolin to blood revealed notable augmentation in blood clotting. Furthermore, the incorporation of kaolin into PVA presented mild diminution in antibacterial activities. Moreover, PVA/Kaolin composites illustrated no cellular toxicity towards fibroblast cells. These remarkable features substantiate that the PVA-K0.1 sponge could be applied as a multifunctional wound dressing.

## Introduction

The leading challenge of injuries treatment is the excessive hemorrhage, which might result in approximately 40% of death, particularly in injuries caused either on the combat zone or accidents^1^. Extreme bleeding could trigger serious and uncontrollable complications; for instance, hypovolemic shock, microbial infections, traumatic death^2^. Hence, prompt management of such bleeding necessitates effective treatments utilizing biocompatible and hemostatic materials. Previous studies put forward that systemic hemostatic and injectable compounds including fibrin, thrombin and growth factors have been implemented to tackle the blood flow in severe patients^3^; however, dangerous consequences have been explored, such as internal clotting and pulmonary embolism^4^. For these reasons, topical clotting materials possess intrinsic hemostatic properties, including as collagen^5^, zeolite^6^ and chitosan^7^ have been drawing great interest.

Blood coagulation mechanism accomplishes in three progressive stages: (1) initiation that includes thrombin development, (2) amplification, in which aggregation and activation of platelets could be perceived, (3) proliferation, which is identified by fibrin construction and steadiness of the platelet clot. The effect of used hemostatic materials commonly implements during the amplification and proliferation stages through constructing a network on the wound site, which promotes the accumulation and clotting of platelets^8^.

Several requisites should be valid in a hemostatic material, involving feasibility to apply, economical, hemocompatible and cytocompatible, and inherently biodegradable^9^. Accordingly, Kaolin has been recognized as being one of the localized potent hemostatic agents that could effectively stimulate the blood clotting process^10^.

Kaolin or china clay is mainly consisting of mineral kaolinite and aluminium silicate^11^. Margolis^12^ found out for the first time the capability of kaolin in blood clotting stimulation. So far, kaolin has been efficiently applied as an active agent for surgical hemostasis since negative charges on the kaolin surface can significantly affect the blood clotting^10^. Within wounds in vivo, once the kaolin placed in direct contact to the blood, it spontaneously prompts the factor XII and platelets to commence the blood coagulation pathway^13^. Moreover, it could be utilized as an analgesic and anti-inflammatory agent during wounds treatment in order to impede the formation of edema.

During the early phases of injuries regeneration, the damaged area of skin usually generates high amounts of exudates, encompassing tissue fluids and dead cells.

Over-secretion of the wound exudates provides an ideal media for microbial growth, instigating harmful infections, which certainly hinder the wound healing^14,15^. Thus, wound dressing candidates should have the ability to accelerate blood clotting^16^, reduce wound inflammation^17^, thwart microbial growth^18^, absorb excess exudates and concurrently conserve the balance of moist onto the wound site^19^. Various antimicrobial wound dressings have been formulated based on several biopolymers supported by antimicrobial compounds^20–23^. Among these materials, hydrogels demonstrated promising properties owing to their three-dimensional network structures, which might be facilely loaded with antibiotics along with their capacities to absorb large amounts of wound exudates^24,25^.

Polyvinyl alcohol (PVA) hydrogel has been receiving numerous interests for implementation in wound management due to its biological outstanding features, comprising biocompatibility, biodegradability, and biosafety to human cells^26^. PVA is a distinctive physical crosslinking hydrogel that could be perfectly crosslinked via repeated freeze-thawing to develop crystalline clusters as a crosslinking point^27^. The shortcomings of the antimicrobial properties and thrombogenicity of PVA could be governed by boosting the hydrogel membranes by certain bioactive compounds, such as antibiotics and kaolin, respectively^28^. Thus, several wound dressing agents incorporating kaolin have been launched, and the most prominent one is QuikClot Combat Gauze (QCCG), which was fabricated from nonwoven gauze impregnated with kaolin; However, main flaws of QCCG are inefficiency to absorb great amounts of wound exudates in addition to the complication to change or remove^16^. By contrast, wound dressings based on the hydrogel are easy and painless to take them out with high capacities to absorb the access of wound exudates, keeping moist at the wound site to enhance the cells migration^19^.

Herein, hemostatic and antibacterial PVA/Kaolin composite sponges with different concentrations of kaolin promoted by penicillin–streptomycin (Pen-Strep) were formulated. Physical and chemical characters of the membranes were investigated to determine the impact of kaolin on PVA. Additionally, the developed membranes were biologically evaluated adopting antibacterial, blood interaction, and cytotoxicity assays.

## Results and discussion

### Fabrication and characterization of PVA/Kaolin membranes

In the current work, a series of PVA/Kaolin sponges were developed through conducting twelve sequential freezing/thawing cycles in order to induce transformation of PVA solution into a crystalline structure, forming an insoluble hydrogel. Accordingly, Non-crosslinked PVA, kaolin, and water molecules were trapped into the physical crosslinked network structure. The prepared hydrogel membranes included different concentrations of kaolin, which were marked as PVA-K0.1, PVA-K0.25, and PVA-K0.5. Furthermore, the addition of Pen-Strep endowed the antibacterial characteristic to the fabricated hydrogels.

### FT-IR analysis

**Figure 1:**
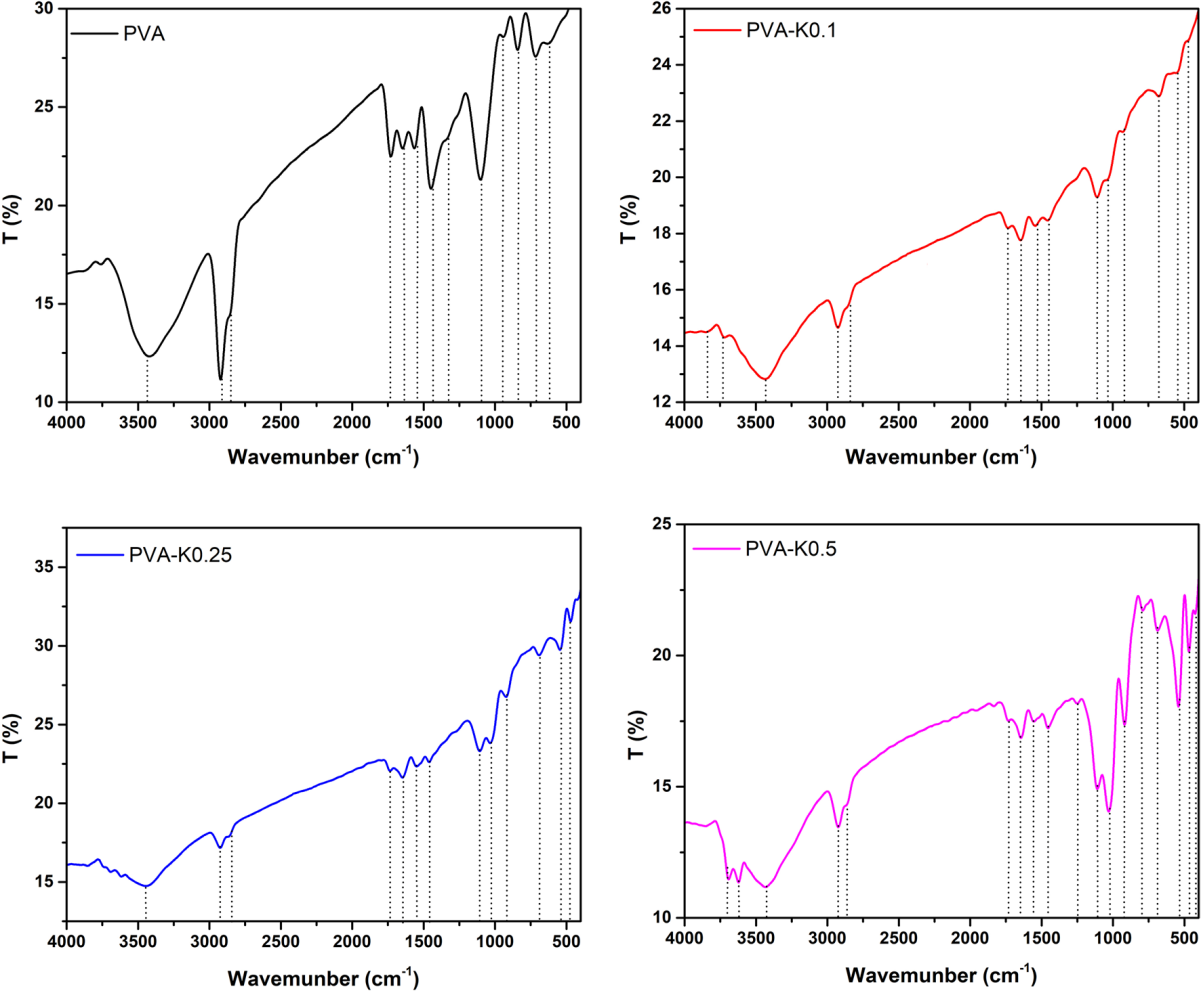
FT-IR spectra of PVA hydrogel membrane and PVA/Kaolin composite hydrogel membranes.

The chemical structures of pure PVA hydrogel and the prepared PVA/Kaolin composite hydrogel membranes, including different concentrations of kaolin were examined by FT-IR as depicted in Fig. 1. It could be recognized from FT-IR spectra the emergence of typical strong –OH bands for free hydroxyl group (–OH) at 3387 cm^−1^ as broadband, which stemmed from hydrogen bonds between –OH groups among PVA chains that endow the hydrophilic forces to PVA hydrogel^29^. PVA spectrum reveals a characteristic peak at 2926 cm^−1^ corresponding to the asymmetrical and symmetrical C-H stretching vibration modes of the methyl groups on the polymer backbone. Furthermore, the peak at 2845 cm^−1^ indicates a methylene vibration band, while the sharp peak at 1710 cm^−1^ is attributed to stretching vibration band of the remain acetyl carbonyl groups in PVA structure. The peak at 1442 cm^−1^ is ascribed to the asymmetrical and symmetrical C–H bending vibrations of methyl group^29^. Moreover, a sharp characteristic peak at 1118 cm^−1^ is a dominant manifestation of the PVA structure^30^, whereas a peak located at 1085 cm^−1^ correlates to C–O–C.

Introduction of kaolin to PVA membranes gave rise to emergence of new peaks in a range from 920 to 940 cm^−1^, which are attributed to Al–OH vibration. Moreover, peaks at 789 and 530 cm^−1^ are ascribed to vibration band of Si–O–Al bonds, while the peak at 470 cm^−1^ is corresponding to Si–O vibration band.

### Thermal characterization

Thermal characteristics of biomaterials intended to medical applications are substantial determinant in order to appraise their commercial implementations^31,32^. Thus, TGA and DSC analyses were adopted to analyze the thermal behavior of PVA/Kaolin composite hydrogel membranes compared with the pure PVA hydrogel membrane.

**Figure 2:**
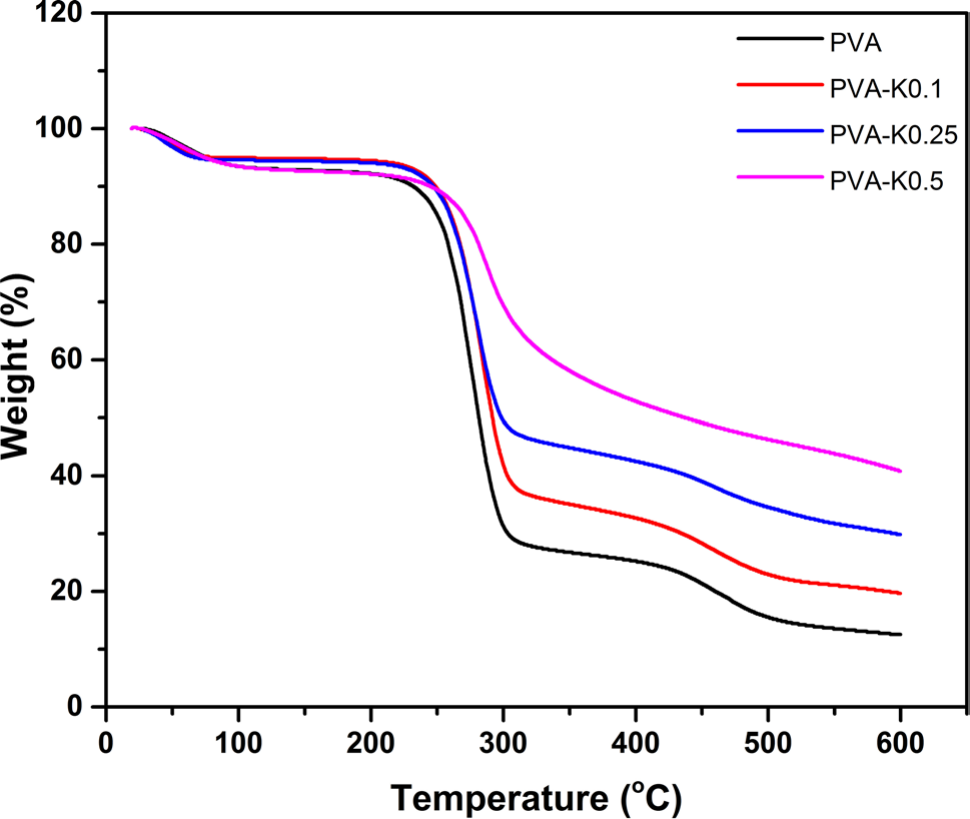
TGA curves of PVA hydrogel membrane and PVA/Kaolin composite hydrogel membranes.

Figure 2 depicts TGA for the PVA and PVA/Kaolin composite hydrogels. All hydrogel samples were exposed to three noticeable weight losses. The first weight decrease for PVA and PVA-Kaolin composites was commenced from ambient temperature to 120 °C, recording 7% of their weights. This is likely imputed to the evaporation of water trapped by hydrophilic hydroxyl groups in the polymer matrix.

The entire hydrogels had a second manifest weight loss in a temperature range from 220 to 320 °C, which might be related to the removal of hydroxyl groups and the formation of polyene macromolecules. These results are in line with those of the previous study^29^. The weight loss level for the PVA membrane was the highest in comparison with the PVA-Kaolin membrane. In details, PVA film was lost 59.27% of the initial weight within a temperature range of 239–307 °C, while the weight of PVA-K0.1 was diminished 53.57% in a temperature range of 226–303 °C. Furthermore, the weight of PVA-K0.25 was decomposed about 46.16% in the temperature range of 226–306 °C, while the degradation percentage of PVA-K0.5 was 26.3% in a temperature range between 241 and 315 °C. It could be evidently recognized that the weight loss rate was reduced with the increase in kaolin concentrations. The alteration of the decomposition peak to lower temperature in case of PVA-K0.1 and PVA-K0.25 is linked to kaolin particles, which distributed inside the PVA matrix and acted as an insulator and mass-transport barrier to the decomposition of hydrogels^30^. By contrast, the behavior of PVA-K0.5 might be attributed to coagulation of kaolin powder, producing larger particles in the PVA matrix.

The third degradation phase was perceived at 600 °C due to the degradation of the formed polyenes. In this stage, the increase in remaining weights from 12.54% for pure PVA to 19.82% for PVA-K0.1, 30.22% for PVA-K0.25 and 40.89% for PVA-K0.5 is related to the stability of inorganic residues of kaolin. Overall, PVA-K0.5 exhibited the best thermal stability, which could be explained by the formation of polyimide foams in the presence of kaolin^33^.

**Figure 3:**
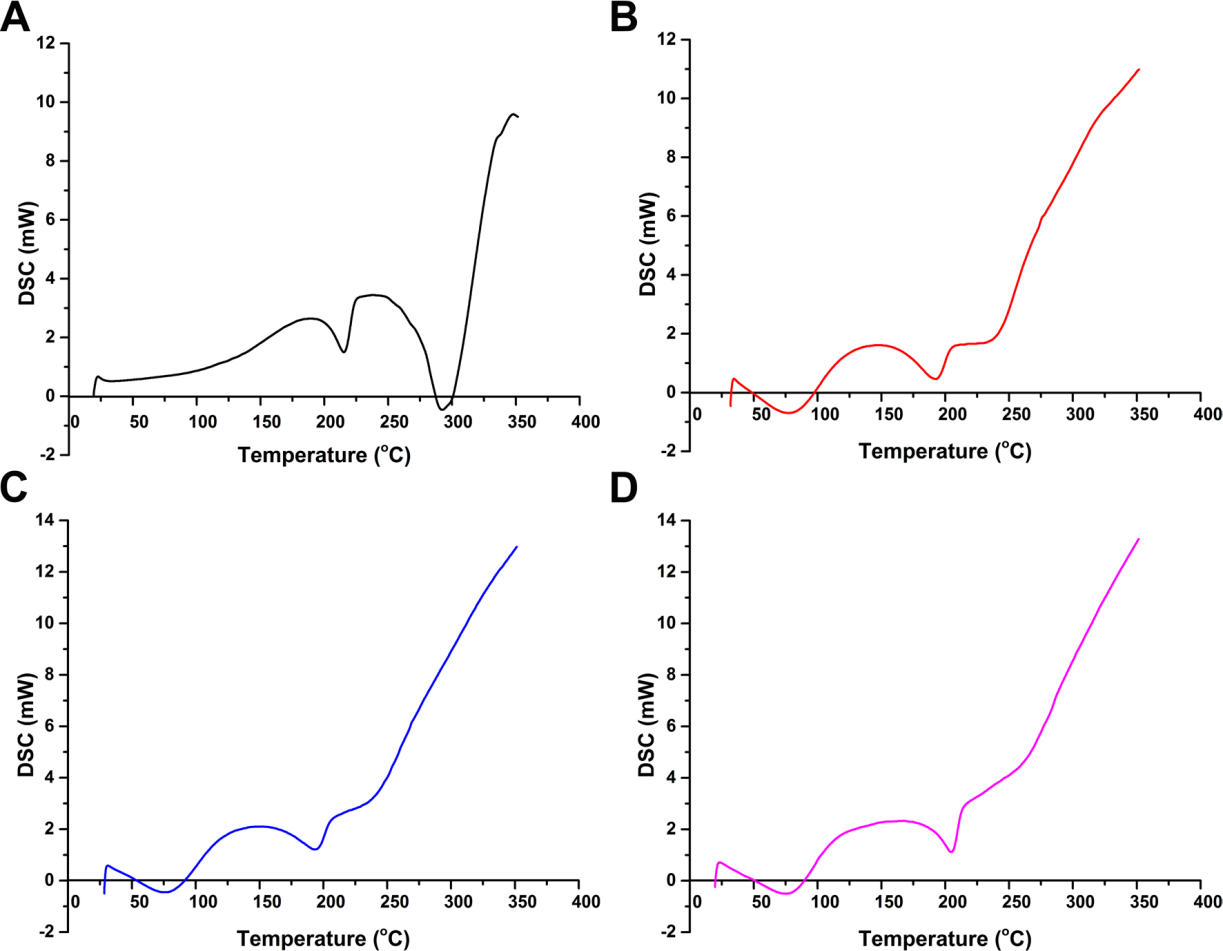
DSC analyses of (**A**) PVA hydrogel membrane, (**B**) PVA-K0.1, (**C**) PVA-K0.25, and (**D**) PVA-K0.5 composite hydrogel membranes.

Figure 3 presents the DSC results for PVA/Kaolin composite hydrogels compared to the prime PVA hydrogel. As can be perceived from the DSC curves, broad endothermic peaks in a temperature range of 70–80 °C, which is ascribed to volatilization of moisture contents trapped in the hydrogel molecules. These findings are in complete agreement with the previous study^34^. The exothermic peaks within a temperature range from 105 to 180 °C could be explained by the relaxation that correlates with the crystalline regions in the hydrogel^34,35^.

The endothermic peaks at 217 °C for PVA, 185 °C for PVA-K0.1, 197 °C for PVA-K0.25 and 199 °C for PVA-K0.5 implies the melting of PVA and distortion of its crystal structure (T_m_), which are consistent with previous results^35^. Furthermore, the shift in T_m_ value of PVA/Kaolin composites to lower temperatures alludes to the crosslinking of PVA and kaolin that influence on the crystal formation^36^. The next exothermic peak is related to thermal decomposition of PVA and elimination of water molecules along with the polymer backbone. It could be deduced from the DSC analyses that the reduction of the peaks by incorporation of kaolin confirms the interaction between volatile decomposition products (water vapour, carbon monoxide, and carbon dioxide) with kaolin particles during the decomposition process that matches with the chain-stripping mechanism^37^.

### Surface morphology of the membranes

The addition of kaolin to PVA might alter the normal internal configurations of PVA molecules that absolutely affect the roughness of their surfaces. Accordingly, the roughness of PVA/Kaolin membranes was significantly augmented from 0.92 ± 0.22 µm in case of PVA hydrogel membrane to 1.03 ± 0.12 µm, 1.2 ± 0.10 µm, and 1.24 ± 0.11 µm for PVA-K0.1, PVA-K0.25, and PVA-K0.5, respectively, as given in Table 1.

**Table 1:** Roughness of PVA hydrogel membrane and PVA/Kaolin composite hydrogel membranes.

Membrane	Roughness (µm)
PVA	0.92 ± 0.22
PVA-K0.1	1.03 ± 0.12
PVA-K0.25	1.20 ± 0.10
PVA-K0.5	1.24 ± 0.11

Values are presented as means ± SD.

**Figure 4:**
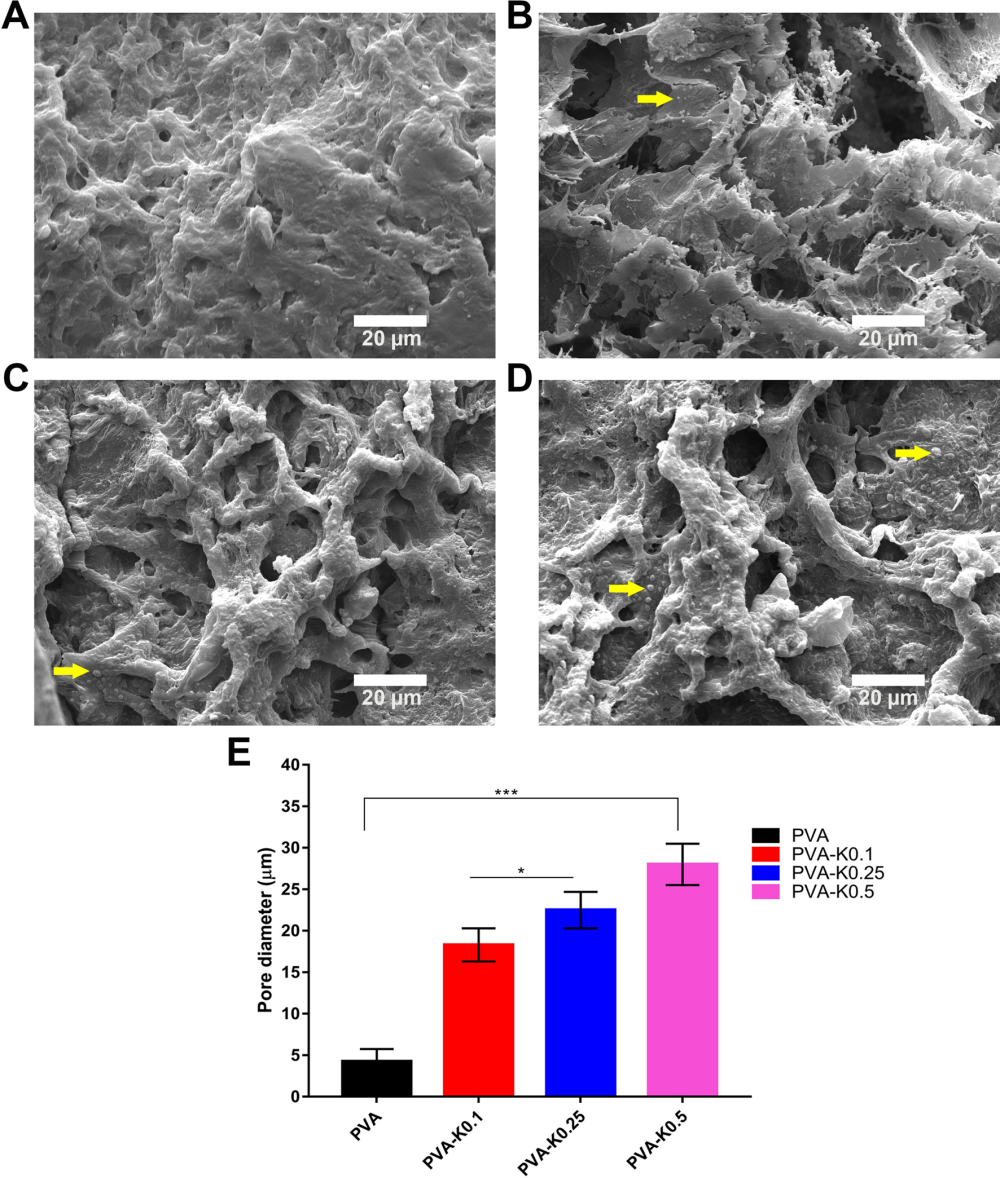
SEM images of surface morphology distinctions of (**A**) PVA, (**B**) PVA-K0.1, (**C**) PVA-K0.25, and (**D**) PVA-K0.5 hydrogels. The arrows point to kaolin particles inside the network of the hydrogels. (**E**) Pores size of PVA and PVA/Kaolin hydrogels. Data are presented as means ± SD (***p < 0.001, and *p < 0.05).

**Figure 5:**
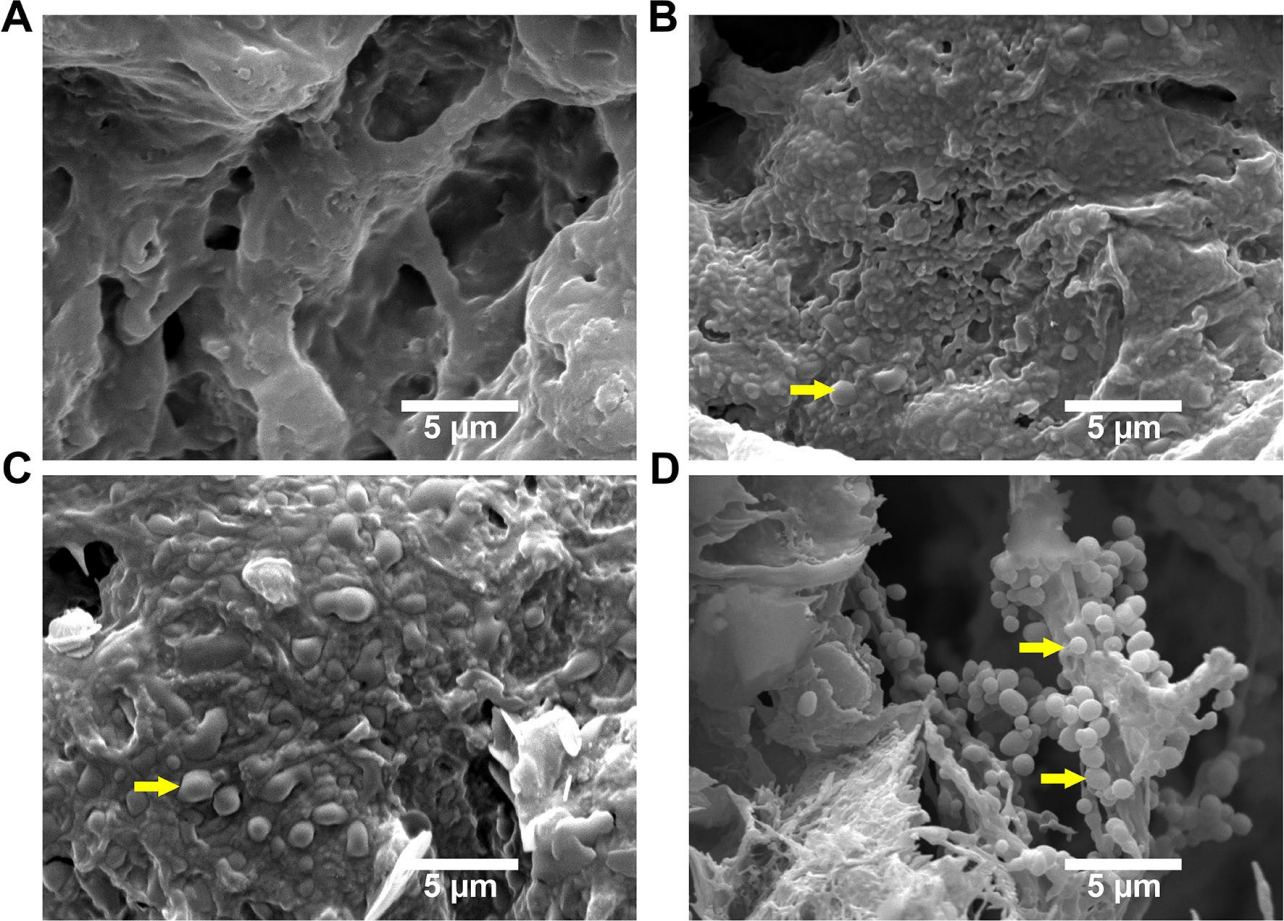
SEM images exhibit cross-sectional morphologies of (**A**) PVA, (**B**) PVA-K0.1, (**C**) PVA-K0.25, and (**D**) PVA-K0.5 hydrogels. The arrows refer to the kaolin particles inside the hydrogels, indicting the variations on the basis of kaolin content.

SEM analysis was conducted in order to discern the morphological changes of PVA/Kaolin composite membranes in comparison with the original PVA membrane. Figure 4 portrays the porosity structures of the formulated PVA and PVA/Kaolin membranes. It can be evidently observed the three-dimensional structure of PVA and the morphological difference for PVA/Kaolin films with different concentrations of kaolin. All the PVA/Kaolin hydrogels demonstrated remarkable porous structures and three-dimensional interconnected pores. Additionally, many entirely dispersed particles of kaolin can be recognized in the matrix of the PVA/Kaolin hydrogels. Moreover, In order to explore the interaction between kaolin particles and PVA, the fracture surface of the PVA/Kaolin composite hydrogels were probed employing SEM in comparison with the pure PVA hydrogel. Figure 5 illustrates the cross-sectional images of the fabricated hydrogels, exhibiting interconnected network with a smooth surface in case of the pure PVA. In contrast, the fractured surfaces for the entire PVA/Kaolin hydrogels were obviously rough, associating with a high aggregation of kaolin particles compared to the unmodified PVA. Moreover, it can be perceived that the aggregation of kaolin over the fractured surfaces augmented with the increase in kaolin concentration, particularly for PVA-K.05. Besides, the lowest concentration of kaolin in PVA-K0.1 was fully connected into the polymer matrices, while the growth of kaolin level gave rise to the materialization of kaolin particles with large size onto the fractured surface of the hydrogel. This could be ascribed to the interfacial interaction between PVA and kaolin alongside the polymer network.

Three-dimensional structure of PVA was previously utilized in preparing porous scaffolds for tissue regeneration and wound healing to enhance the attachment and proliferation of dermal fibroblasts^38^. Moreover, the porous structure of the membrane could stimulate the absorption of wound surplus exudates^13^, enable diffusion of vital nutrients for the cells^24^, and allows the exchange of gases and fluids at the wound site.

It is apparent that the pore sizes of PVA/Kaolin hydrogels are larger than those for pure PVA with average diameters of 29 μm for PVA-K0.5. Precisely, the average pore diameters of PVA, PVA-K0.1, PVA-K0.25, and PVA-K0.5 were 4.5 ± 1.5 µm, 18.4 ± 2.3 µm, 22.5 ± 2.2 µm, and 29 ± 2.9 µm, respectively, as illustrated in Fig. 4E. This could be ascribed to the reduction in crosslinking density of the hydrogels with the increase of kaolin content in the PVA matrix^39,40^. Moreover, recent studies demonstrated the significant decrease in pore sizes with the rise of the crosslinker concentration in the hydrogels^41^.

These results are in line with those of previous reports^39^, which formulated effective hydrogels for wounds treatment with pore sizes ranging from 19.5 to 36.7 μm. Moreover, previous studies demonstrated that pores with average diameter between 20 and 125 μm of wound dressings had a pivotal role in skin regeneration through supplying oxygen and required nutrients for the cells^42^. Specifically, during the wound healing process, prior investigations reported that the pore sizes within range of 3–20 μm ameliorated the proliferation of epidermal keratinocyte and dermal fibroblast cells since they could promote cell–cell communication and distribution of various soluble nutrients either from a culture medium or wound substrate^43^.

Taken together, the SEM results pointed out that the morphological structure of PVA/Kaolin membranes could be varied on the basis of kaolin concentrations, which could be attributed to the rearrangement of kaolin particles inside PVA matrix.

### Gel fraction, swelling behavior, porosity, and in vitro degradation

The impact of various kaolin contents on gel formation was examined. It could be extrapolated from the data that increase in incorporated kaolin level in PVA resulted in a decrease in gel fractions as presented in Table 2. In case of PVA, the gel fraction was 87.6 ± 4.4%, whereas the gel fractions were determined to be 84.8 ± 4.2%, 83.1 ± 4.2%, and 78.8 ± 3.9% for PVA-K0.1, PVA-K0.25, and PVA-K0.5, respectively. This manner is likely to be related to the distortion effect of kaolin on the PVA crystal structure. These findings imply that PVA was almost completely crosslinked in the absence of kaolin^44^, while the kaolin lessened the crosslinking, improving the swelling features of PVA/Kaolin sponges. This enables the absorption of blood and wound exudates upon applying the prepared sponges. Additionally, the decrease in gel fraction associates with weakness of flexibility and gel strength. These results match those observed in earlier investigations^45,46^.

**Table 2:** Gel fractions of the PVA hydrogel membrane and PVA/Kaolin composite hydrogel membranes.

Membrane	Gel fraction (%)
PVA	87.6 ± 4.4
PVA-K0.1	84.1 ± 4.2
PVA-K0.25	83.1 ± 4.2
PVA-K0.5	78.8 ± 3.9

Data are presented as means ± SD.

**Figure 6:**
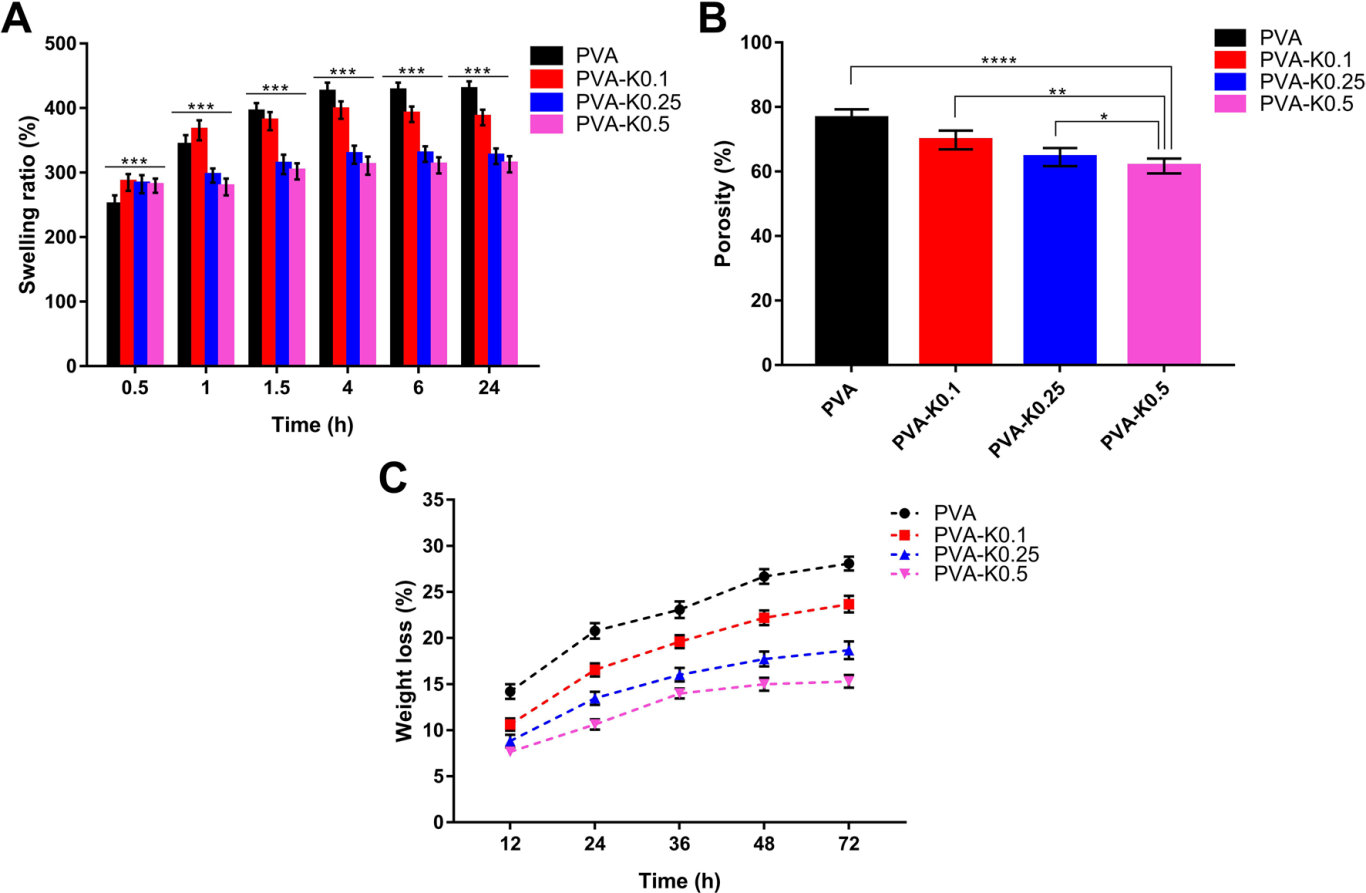
(**A**) Swelling ratio, (**B**) porosity, and (**C**) in vitro degradation for PVA/Kaolin composite hydrogel membranes in comparison with PVA hydrogel membrane. Data are presented as means ± SD (***p < 0.001, **p < 0.01, and *p < 0.05).

It is well known that the spongy-like structure of hydrogels along with their hydrophilic groups can promote the swelling properties of the hydrogels^47^. In vitro swelling behavior of the PVA/Kaolin hydrogel composites was evaluated as illustrated in Fig. 6A. Swelling ratio investigation exhibited a significant difference with p < 0.001 in the swelling ratios of the PVA-K0.1, PVA-K0.25 and PVA-K0.5 films compared with PVA group. Rapid swelling was perceived for PVA/Kaolin hydrogel membranes within 30 min in comparison with PVA. Moreover, the highest water absorption of 365 ± 15% was found for PVA-K0.1 after 1 h among the other tested membranes. This might be stemmed from the rise in the pore sizes as a consequence of kaolin incorporation, which is in complete agreement with the SEM results. It could be also perceived that the entire groups attained a swelling equilibrium in a period of 4 h. Furthermore, PVA-K0.5 attained the lowest water absorption of 310 ± 15% within 4 h, whereas pure PVA, PVA-K0.1, and PVA-K0.25 reported water absorption of 424 ± 17%, 397 ± 12%, and 327 ± 14%, respectively. The comparable results were obtained after 24 h of immersing in water. These outcomes manifestly indicate that the swelling ratio lessened with increasing the concentrations of kaolin in PVA/Kaolin hydrogels. Although these diminutions of water uptake ratios, previous studies demonstrated the in vivo effective performances of devised wound dressings based on PVA and other biomaterials with swelling ratios of 102% and 130%^22,48^. Significantly, this performance is supportive of cells adherence and proliferation since the water uptake capability is sensible to the cell infiltration, thus a great water uptake enables the permeation of cells inside the three-dimensional structure hydrogels^21,49^. Altogether, the water uptake capabilities of PVA/Kaolin emphasize that these wound dressings can absorb the surplus of wound exudates and thus thwart the microbial infections, proposing PVA-K0.1 as the best wound dressing candidate.

The porosity of the fabricated composite hydrogels was investigated to evaluate their water holding capacity. As demonstrated in Fig. 6B, the incorporation of kaolin decreased the porosity of the membranes in comparison with pure PVA membrane. Precisely, the porosity for PVA was 76 ± 3%, while PVA-K0.1, PVA-K0.25, and PVA-K0.5 recorded porosity of 70 ± 4%, 64 ± 3%, and 62 ± 2%, respectively. These observations are corresponding to the previous report^50^, and are likely due to the occupancy of hydrogel pores by kaolin particles, which make them more compacted and frustrate parts of previously accessible pores. The great porosity alongside the water uptake capacity of wound dressings are favorable to hamper the microbial infections, increase the surface area for drug loading, and promote the cells proliferation, which thus boost the wound healing process through reducing the required duration, particularly inflammation stage.

With respect to the in vitro degradation, the degradation characteristic of wound dressings is paramount for implementing their biological functions efficiently; for instance, the release of loaded drugs can be influenced by the degradability of membranes. Thus, the weight loss of PVA/Kaolin membranes was estimated in vitro using PBS buffer (pH 7.4) at 37 °C for different time points. After 72 h of incubation in PBS, all membranes had noticeable weight losses of 28 ± 0.8%, 24 ± 0.6%, 19% ± 0.7, and 15 ± 0.5% for PVA, PVA-K0.1, PVA-K0.25, and PVA-K0.5, respectively, as displayed in Fig. 6C. These data expose the good biodegradation features of the formulated hydrogels.

Collectively, water uptake, porosity, and biodegradation properties of PVA/Kaolin composite membranes suggest PVA-K0.1 as the potent wound dressing membranes among the other groups.

### Adhesive strength evaluation

Adhesive capability is a crucial feature for hemostatic hydrogels^51^; therefore, Influence of kaolin on the adherence properties of PVA/Kaolin hydrogels was studied. The results exhibited a significant increase of adhesive strength ability from 33.5 ± 1.7 N cm^−2^ for PVA membrane to 40.18 ± 2 N cm^−2^ for PVA-K0.1. However, this value was dramatically decreased with the increase in kaolin content, recording 30.27 ± 1.5 N cm^−2^ and 27.23 ± 1.4 N cm^−2^ for PVA-K0.25 and PVA-K0.5 groups, respectively.

Adhesion of PVA hydrogel membrane is mainly related to the hydrogen bonds existed in its function groups. Furthermore, addition of small amount of kaolin in case of PVA-K0.1 led to increase in the hydrophilicity of the hydrogel, improving the adhesion capability. On the contrary, rise in kaolin concentration into hydrogels might result in significant alterations of the dense network and mesh structure, provoking the lessening of adhesion properties^52^. Collectively, these findings emphasize the improvement of PVA-K0.1 adhesion as a good candidate for wound dressing.

## Biomedical evaluations

### Antibacterial activity

**Figure 7:**
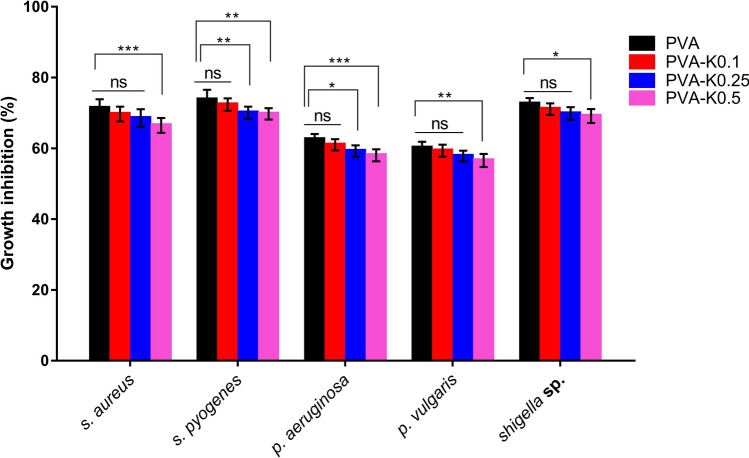
Antibacterial assays of PVA hydrogel membrane and PVA/Kaolin composite hydrogel membranes loaded with Pen-Strep against *S. aureus*, *S. pyogenes*, *P. aeruginosa*, *P. vulgaris*, and S*higella* sp. Data are presented as means ± SD (***p < 0.001, **p < 0.01, and *p < 0.05).

One of the substantial properties of wound dressing materials is their competence to impede the microbial infections that accelerate the wound healing and further skin remodeling^53,54^. Hence, we boosted the formulated hydrogel membranes by Pen-Strep, which is conventionally applied during propagation of various kinds of cell lines due to its safety and performance to hamper the microbial contaminations. Antibacterial behaviors of the devised membranes against *P. aeruginosa*, *Shigella* sp., *P. vulgaris*, *S. aureus*, and *S. pyogenes* were evaluated as given in Fig. 7. All membranes nearly revealed comparable antibacterial trends against the tested bacteria. Moreover, it could be perceived that the PVA film exerted the greatest growth inhibition towards the entire bacteria due to the effect of Pen-Strep. The first glance points out that the antibacterial performances of membranes were slightly decreased by the addition of kaolin at all levels. Nevertheless, there were no significant differences in the growth inhibition for PVA-K0.1 and PVA in relation to the entire bacterial strains. In contrast, the bacterial growth inhibition of *S. aureus*, *P. vulgaris*, and *Shigella* sp. quite exhibited no significant differences of PVA-K0.25 and PVA hydrogel membranes, while significant statistical differences were found out in case of *S. pyogenes*, and *P. aeruginosa*. Moreover, the highest and significant differences were recognized in a two-way ANOVA for PVA-K0.5 and PVA with regard to the whole bacteria. These manners might be elucidated by the influence of kaolin in adsorption of Pen-Strep molecules, trapping them inside the hydrogel and consequently hinder their release. However, this action cannot persist since the fast degradation of the hydrogel network completely released the Pen-Strep and kaolin into the media.

These results are absolutely supported by the in vitro degradability data of the fabricated hydrogels and thus highlight the selection of PVA-K0.1 wound dressing.

### Evaluation of blood and hydrogel membranes interactions

**Figure 8:**
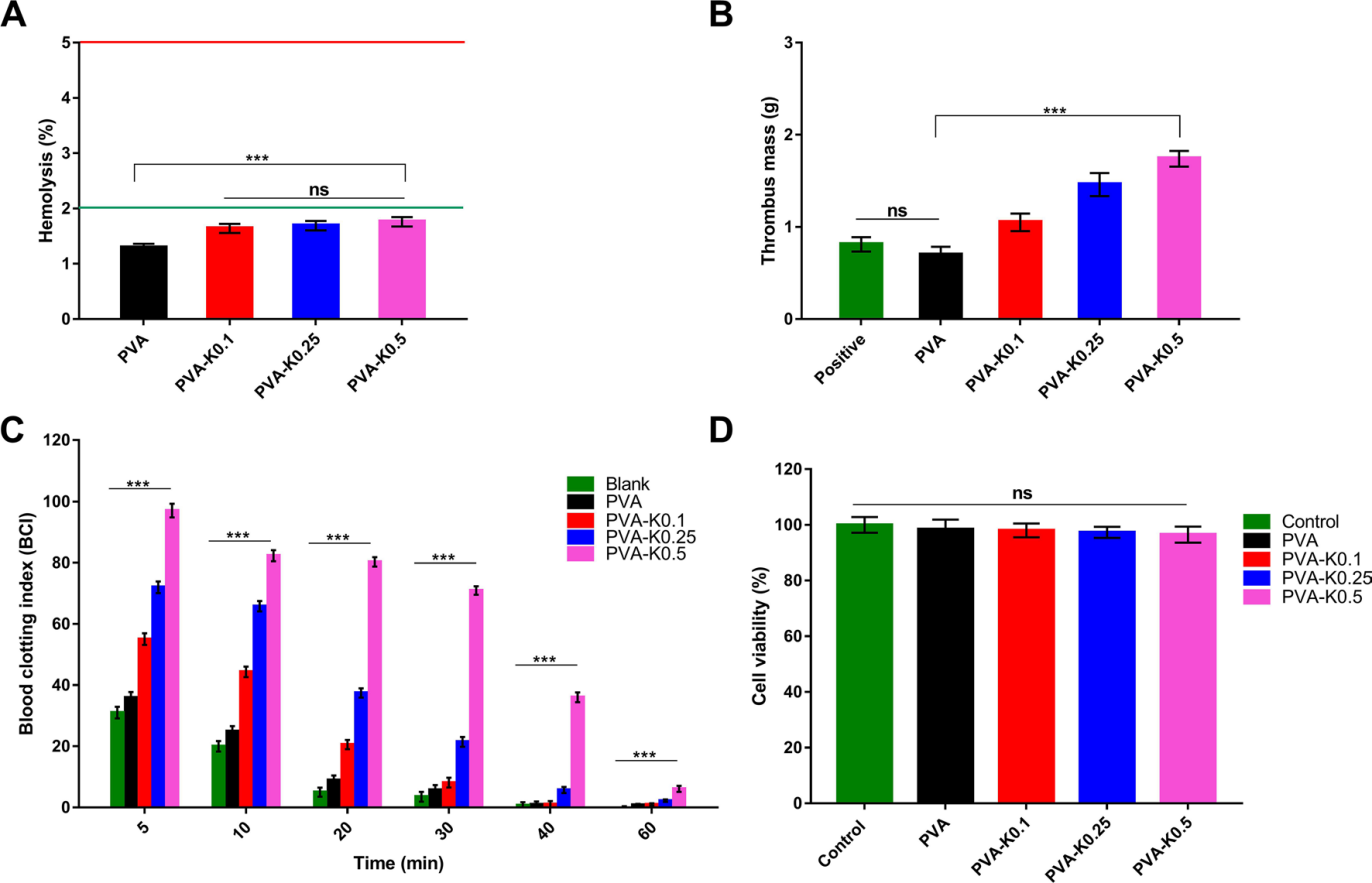
(**A**) Hemocompatibity, (**B**) thrombogenicity, (**C**) blood clotting index, and (**D**) cytotoxicity for PVA hydrogel membrane and PVA/Kaolin composite hydrogel membranes. Data are expressed as means ± SD [***p < 0.001, while (ns) indicates non-significant difference].

Blood compatibility is one of the underlying properties required in the wound dressings^55^. Hemocompatibility of PVA and PVA/Kaolin composite hydrogel membranes was measured to investigate the prospect to trigger hemolysis of RBCs. Figure 8A demonstrates the hemolytic percentages of the studied membranes. The findings showed no significant variances of hemolysis between the PVA supported by different concentrations of kaolin. However, the statistical analysis demonstrated significant differences in PVA compared with the PVA/Kaolin hydrogels. Although these differences, the PVA/Kaolin membranes presented slight hemolysis less than 2%, which considers the safe level according to ASTM.

The thrombogenicity of PVA and PVA/Kaolin membranes was examined as shown in Fig. 8B. PVA membranes showed a lower tendency for thrombus formation than that of blood control because of the hydrophilic nature of PVA. By contrast, the addition of kaolin to PVA membrane showed a significant augmentation in thrombus formation that could be certainly attributed to the activity of kaolin as an active clot agent.

In order to evaluate the ability of the formulated membranes to accelerate the hemostatic process, the clotting index of PVA and PVA/Kaolin composite membranes at time intervals was examined as illustrated in Fig. 8C. It could be obviously recognized the role of kaolin in the acceleration of clotting formation. The increase in kaolin contents evidently enhanced blood clotting index. We presume the presence of both hydrophobic properties and clotting ability of kaolin resulted in fast adsorption of a blood protein that is responsible for the initiation of the clotting process^13^.

### Cytotoxicity evaluation

Cytotoxicity assay of wound dressing candidates is a decisive approach to assert whether the studied materials could be further considered for extensive in vitro and in vivo evaluations. This step is indispensable as the wound dressing agents will be directly contacted with fibroblast, keratinocyte and epithelial cells during the wound healing^17,22^. Thus, the cellular response in presence of the fabricated membranes was evaluated utilizing fibroblasts as relevant cells at the wound bed, which are responsible for tissue granulation through the generation of connective tissue to enhance the skin regeneration. The present findings revealed no toxicity of the membranes against fibroblasts.

The increase of kaolin levels inside the membranes mildly diminishes the cell viabilities as depicted in Fig. 8D. The lowest cell viability reached 96.5% for PVA-K0.5; however, it is still inconsiderable and the statistical analysis demonstrated no significant difference of the tested hydrogel membranes compared to the untreated cells. It could be inferred from these results that there is no toxicity of the membranes, demonstrating the suppression of undesirable upshots over the wound recovery in vivo. Manifestly, these intriguing findings strongly propose the further in vivo evaluations of the PVA-K0.1 hydrogel composite membrane.

## Conclusion

In conclusion, hemostatic and antibacterial wound dressing PVA/Kaolin hydrogel membranes supplemented with penicillin–streptomycin were developed and characterized. Furthermore, the influence of kaolin concentrations on the physicochemical and biological properties of PVA/Kaolin hydrogel composites was studied. SEM analysis revealed a significant increase in the pores size of the PVA/Kaolin hydrogel membranes. Besides, the addition of kaolin enhanced the swelling capacities of the fabricated composites over the first 30 min and augmented the pores size of the fabricated hydrogels, which promote the absorption of wound exudates. Furthermore, high porosity and good biodegradation were found for PVA/Kaolin composites. Nevertheless, the adhesive strength was exclusively improved for PVA-K0.1 group. The antibacterial evaluation proved the expected action of the supported antibiotic, which imparted the antibacterial potency towards the membranes. Moreover, the entire membranes exhibited hemocompatibility in addition to their capability to clot the blood as a consequence of kaolin incorporation. The cellular toxicity corroborated the cytocompatibility of the membranes towards the fibroblast cells. Our findings clearly proposed PVA-K0.1 as a potent hemostatic and antibacterial wound dressing among the other groups. Accordingly, we are currently in the process of extending the investigation of this multifunctional composite in vitro and in vivo to utilize as a favorable wound dressing.

## Materials and methods

### Materials and bacterial strains

PVA (typically average M_w_ = 72,000 g mol^−1^; 98.9% hydrolyzed) was supplied by Biochemica, Germany. Kaolin, MTT 3-(4,5-dimethylthiazol-2-yl)-2,5-diphenyltetrazolium bromide, sodium hydroxide, ethanol, dimethyl sulfoxide (DMSO), and acid citrate dextrose solution (ACD) were purchased from Sigma-Aldrich (Chemie GmbH, Steinheim, Germany). Penicillin–streptomycin (Pen-Strep) (10 KU/10 KU) was obtained from Lonza, Belgium. Yeast extract and tryptone were procured from Bioshop (Canada Inc.). Sodium chloride was provided by Adwic Co. (Egypt).

Gram-negative [*Pseudomonas aeruginosa* (*P. aeruginosa*), *Shigella* sp., and *Proteus vulgaris* (*P. vulgaris*)] and Gram-positive [*Staphylococcus aureus* (*S. aureus*), and *Streptococcus pyogenes* (*S. pyogenes*)] bacteria were used to investigate the antibacterial activities of the formulated materials. The strains were revitalized from glycerol vials by growing overnight at 37 °C and 150 rpm into Luria Bertani (LB) broth medium, consisting of NaCl 10 g l^−1^, peptone 10 g l^−1^, and yeast extract 5 g l^−1^.

#### For blood tests

Informed consents were obtained from all volunteers before the use of their blood for hemocompatibility, thrombogenicity, and blood clotting evaluations. This research was approved by the ethical local committee at the General Authority of the City of Scientific Research and Technological Applications, Ministry of Scientific Research, Egypt and New Borg El-Arab hospital, Alexandria, Egypt. Moreover, the entire analyses were performed in accordance with relevant guidelines.

### Methodology

#### Preparation of membrane

Aqueous solutions of 5% PVA was prepared by dissolving pre-weighed quantities of PVA powder in distilled water with heating. Next, the PVA solutions were cooled to room temperature, and then different concentrations of kaolin (0.1, 0.25, and 0.5 w/w) in addition to Pen-Strep were added with vigorous stirring at 25 °C. Afterwards, the mixtures were stirred and ultrasonicated to obtain homogeneous composite solutions. The PVA/Kaolin solutions were then poured into plastic Petri dishes, which were further placed in a freezer at − 4 °C for 16 h to induce crystallization. Thereafter, the Petri dishes were taken out and the blends were thawed at room temperature for 8 h. The freezing/thawing cycle was conducted for twelve times. The hydrogels incorporated with 0.1, 0.25 and 0.5 (w/w) of kaolin were labelled as PVA-K0.1, PVA-K0.25, and PVA-K0.5, respectively, alongside PVA as a control. The obtained hydrogel membranes were frozen in liquid nitrogen for 10 min and subsequently lyophilized for further characterizations.

### Characterization of membranes

#### FT-IR analysis

The chemical structures of the fabricated PVA/Kaolin membranes were examined by mixing each sample (~ 5 mg) thoroughly with potassium bromide (KBr), and then analyzed by means of Fourier transform infrared spectrophotometer (Shimadzu 8400S, Japan). The FT-IR instrument was set up in order to scan the sample for 40 scans at a range of 400–4000 cm^−1^.

#### TGA and DSC analyses

To study the thermal properties of the membranes, ∼ 5 mg of the investigated materials were integrated into an aluminium pan and the analysis was carried out employing thermal gravimetric analyzer (Shimadzu 50/50H, Japan) at a temperature range from 20 to 600 °C with a heating rate of 10 °C min^−1^ under nitrogen flow (30 ml min^−1^)^56^.

DSC analysis of membranes was implemented using a DSC instrument (Shimadzu 60A, Japan). The scanning analysis was conducted in a range of 30–350 °C under a nitrogen atmosphere at a flow rate of 30 ml min^−1^ and a heating rate of 10 °C min^−1^^57^.

#### Investigation of surface morphology

To inspect the microstructure and the morphological alterations of the membranes, each sample was coated under vacuum with a thin layer of gold and then examined employing scanning electron microscope (Joel Jsm 6360LA, Japan)^58^.

The surface roughness of the membranes was assessed by means of a surface roughness tester (SJ-201P, Japan). Membranes with Dimensions of 25 mm × 25 mm were fixed onto a glass slide with double-sided tape and the entire measurements were implemented in six replicates.

#### Gel fraction analysis

The concentration of the crosslinked material, forming the insoluble fraction was estimated following ASTM D 2765-01 with some adjustments. The Hydrogels were packaged in stainless steel 500 mesh porous bag. After drying in an oven at 60 °C until a constant weight was obtained, each sample was submitted to extraction employing a soxhlet system using distilled water as a solvent. After 4 h, the bags were dried and re-weighed until constant weights were recorded. The gel fraction was analysed using six replicates of each formulation. The gel fraction was estimated using Eq. (1):

1 $${\text{Gel }}\left( \% \right) = \left( {{\text{Wd}}/{\text{Wi}}} \right) \times {1}00$$

where (Wi) is the initial weight of the dry gel, and (Wd) is the weight of the extracted dry gel.

#### Swelling test

To assess the swelling properties of the developed hydrogels at room temperature, reverse osmosis water was employed. Each sample was weighed and then submerged in water for predetermined times. Then, the samples were removed, carefully blotted using filter papers, and weighed. The swelling test was performed for time intervals up to 24 h. All determinations of each formulation were replicated six times. The swelling percentages of hydrogels were calculated using Eq. (2):

2 $${\text{Swelling }}\left( \% \right) = [({\text{Ws}}{-}{\text{Wd}})/{\text{Wd}}] \, \times {1}00$$

where (Ws) is the weight of the swollen gels, while (Wd) refers to the weight of gels before immersion in water.

#### Porosity measurement

The porosity of the developed hydrogel membranes was estimated following the procedure described by Yin et al.^59^. The entire samples were dried in a vacuum oven at 50 °C for 2 h. Subsequently, the initial weights of the different samples were determined prior to immersing in absolute ethanol for 4 h. The swollen hydrogels were weighed after blotting the redundant ethanol over their surfaces by filter papers. The experiments were performed in six replicates and the porosity was evaluated using the Eq. (3):

3 $${\text{Porosity }}\left( \% \right) = \left[ {\left( {{\text{W2}} - {\text{W1}}} \right)/p{\text{V}}} \right] \times {1}00$$

where W1 and W2 are the weight of the hydrogel prior and after immersing in ethanol, respectively, “V” is the volume of the hydrogel, and “*p*” is the density of absolute ethanol.

#### In vitro degradation evaluation

Initially**,** dried films were weighed and then immersed into 3 ml of 0.1 M phosphate buffer saline (PBS, pH 7.4) at 37 °C for time intervals. Next, the samples were taken out and gently blotted by a soft paper to eliminate the water over their surfaces. Finally, the membranes were dried under vacuum conditions at room temperature and reweighed. All investigations were carried out in six replicates.

#### Adhesive strength evaluation

The adhesive strength of the PVA hydrogels was measured on the surface of the glass plate according to ASTM D-903 by a universal testing machine (Shimadzu AG-1S, Japan)^52^. The length, width and thickness of the tested samples were 100 mm, 20 mm and 6 mm, respectively. As a hard backing of the hydrogel, double phase adhesive tape was used to adhere the hydrogel to the glass plate. The peeling examination was performed at a speed rate of 200 mm min^−1^. For each sample, the test was replicated for six times.

## Biomedical evaluations

### Antibacterial assay

Antibacterial activity of the developed membranes was conducted against pathogenic bacteria by adapting the previous procedures^60,61^. The overnight bacterial cultures were diluted in LB medium and the turbidities were amended in accordance with the McFarland 0.5 standard at 625 nm and the colony-forming unit of the bacterial cultures were 1–2 × 10^8^ CFU ml^−1^. Afterwards, 100 µl of the bacterial suspension was cultured into 10 ml of LB medium, containing 100 mg of hydrogel membrane, while the bacterial cultures free of membranes were served as a control. The inoculated cultures were then grown at 37 °C and 150 rpm for 18 h. The bacterial growth inhibition rate was then gauged by measuring the turbidity at 600 nm. The antibacterial studies were implemented in six replicates and the bacterial growth inhibitions were assessed following Eq. (4):

4 $${\text{Bacterial growth inhibition }}\left( \% \right) = \left[ {\left( {{\text{ODc}} - {\text{ODs}}} \right)/{\text{ODc}}} \right] \times {1}00$$

where ODc and ODs are the optical densities of a bacterial culture without and with a tested membrane, respectively.

### Evaluation of hemocompatibility

The blood was collected from volunteers after obtaining their consents to evaluate the hemocompatibility of the fabricated membranes as previously reported^62^.

To find out the hemocompatibility of the examined membranes, the proposed hemolysis test by the American Society for Testing and Materials (ASTM) (ASTM F 756-00, 2000) was conducted with slight adaptations. Initially, 9 ml of the obtained blood was gently poured into a tube including 1 ml of anticoagulant acid citrate dextrose solution (ACD). Prior to contacting the samples to the blood, each membrane (1 cm^2^) was submerged in a test tube, containing 7 ml of phosphate buffer solution (PBS) pH 7.0 at 37 °C for 72 h. Following this, the PBS was discarded before immersing the samples in 1 ml ACD blood and then kept at 37 °C for 3 h.

Negative and positive controls were set by adding the equal volume of ACD blood to 7 ml of PBS and water, respectively. To conserve the contact of the examined materials to the blood, the tubes were slightly inverted twice each 30 min. Finally, the blood and diluted blood were transferred to new tubes and clarified by centrifugation at 2000 rpm for 15 min. The liberated hemoglobin as a consequence of blood hemolysis was determined by means of a spectrophotometer (Model Ultrospec 2000) at 540 nm. The blood hemolysis experiments were replicated six times under the identical conditions, and the percentage of hemolysis was calculated using Eq. (5):

5 $${\text{Blood hemolysis }}\left( \% \right) = \left[ {\left( {{\text{ODs}} - {\text{ODn}}} \right)/{\text{ODp}} - {\text{ODn}}} \right] \times {1}00$$

where ODs is the optical density of a tested sample, ODn refers to the optical density of the negative control, and ODp is the optical density of the positive control.

### Thrombogenicity test

The development of thrombus on the surface of the developed membranes was appraised adopting a gravimetric method as previously proposed^63,64^. ACD blood was prepared as described above in the hemolysis test. Initially, membranes were submerged into PBS and incubated at 37 °C for 48 h. Then, the PBS was taken out and the ACD rabbit blood was applied over the surface of the studied materials, whereas a positive control was prepared by introducing the same amount of blood to an empty Petri dish. For inducing the blood clotting reaction, 20 µl of a 10 M calcium chloride solution was added onto the membrane. The reaction was ceased after 45 min by adding 5 ml of H_2_O. Eventually, the clots were fixed through applying 5 ml of a 36% formaldehyde solution, and then dried with tissue paper and finally weighed. Each determination was performed in six replicates.

### Blood clotting test

Influence of the prepared hydrogel membranes on blood clotting was studied following a previously published protocol with minor changes^65^. A series of the tested sponges with size 8 mm × 8 mm was positioned at the bottom of 100 ml beakers and then pre-warmed at 37 °C for 5 min, while cotton gauze was used as a blank. ACD blood was mixed with 0.2 M CaCl_2_ at a ratio of 10:1, respectively. Then, 0.25 ml of the recalcified blood was instantly dropped over the surface of the examined membranes and then incubated for 5 min at 37 °C. Afterwards, 10 ml of distilled H_2_O was sensibly poured onto the membranes to release the erythrocytes, which were not trapped inside the clotted blood. A reference value represents the unclotted blood lysed by H_2_O was prepared by adding 10 ml of H_2_O to 0.25 of blood. The absorbance of the liberated haemoglobin was determined by means of a spectrophotometer at 540 nm. The blood clotting examination was conducted in six times and the blood clotting index (BCI) was quantified by the following Eq. (6):

6 $${\text{Blood clotting index }}\left( {{\text{BCI}}} \right) = \left[ {\left( {{\text{Ah}} - {\text{Am}}} \right)/{\text{Ah}}} \right] \times {1}00$$

where Ah, and Am are the absorbance of blood lysed by H_2_O and the released haemoglobin post contacting to the membrane, respectively.

### Cytotoxicity assay

The cellular toxicity of the developed membranes was examined towards mouse fibroblast cell line (NIH 3T3) employing MTT [3-(4,5-dimethythiazol-2-yl)-2,5-diphenyltetrazolium Bromide] assay as described earlier^66,67^. The fibroblast cells were propagated in Dulbecco’s modified Eagle’s medium (DMEM) complemented with 10% fetal bovine serum and nurtured at 5% CO_2_ and 37 °C with a humidity of 85% in a CO_2_ incubator. At the confluence of cells approximately 85%, the cells were detached and then seeded at a density of 2 × 10^4^ cells/well in a 96-well plate. Then, 20 mg of each membrane was exposed to UV for 45 min and applied onto the cells, while the wells free hydrogel were served as control cells. After that, the 96-well plate was maintained for 48 h, and the cells were subsequently washed with PBS thrice to eliminate the residual of materials. Afterwards, each well was provided with 20 µl of MTT solution (5 mg ml^−1^ in serum-free medium) and 180 µl of the respective medium, and the plate was then incubated for 4 h at 37 °C. Thereafter, the media were aspirated and 200 µl of dimethylsulfoxide (DMSO) was added into each well to dissolve the formazan crystals. The plate was thoroughly agitated at 120 rpm for 3 min, and the absorbance was measured at 570 nm using a microtiter plate reader. The cytotoxicity assay was replicated six times, and the viability of the fibroblast cells was determined using Eq. (7):

7 $${\text{Cell viability}} = \left( {{\text{Am}} - {\text{Ac}}} \right) \times {1}00$$

where (Am) indicates the absorbance of cells treated with a tested membrane, while (Ac) is the absorbance of untreated cells.

### Statistical analysis

All investigations were performed in six replicates, and the obtained data were statistically analyzed using GraphPad Prism software (Version 5). One-way and two-way analyses of variance (ANOVA) with Tukey’s test for multiple comparisons were adopted to estimate significant results. All values are presented as means ± SD and the results were considered as statistically significant at p value < 0.05, where n = 6.
